# Factors Influencing Immunotherapy Response in Neuroblastoma: From Tumor Microenvironment to Combination Strategies

**DOI:** 10.3390/cells15050441

**Published:** 2026-02-28

**Authors:** Xiaoran Du, Rui Dong, Kuiran Dong

**Affiliations:** 1 Department of Pediatric Surgery, Children’s Hospital of Fudan University, Shanghai 201102, China; duxiaoran2021@163.com; 2 Shanghai Key Laboratory of Birth Defect, Children’s Hospital of Fudan University, Shanghai 201102, China

**Keywords:** neuroblastoma, tumor microenvironment, immunotherapy, combination strategies

## Abstract

**Highlights:**

**What are the main findings?**

- This review systematically deconstructs the multifaceted determinants of immunotherapy response in neuroblastoma into four interconnected domains—tumor microenvironment (TME), tumor cell-intrinsic properties, host genetic background, and treatment-induced adaptive resistance—revealing that therapeutic outcome is governed by a dynamic ecosystem rather than any single factor.
- We synthesize emerging evidence from single-cell and spatial multi-omics studies demonstrating that therapy-driven adrenergic-to-mesenchymal (ADR-MES) lineage plasticity, coupled with spatiotemporal remodeling of an immunosuppressive TME (SPP1+ TAMs, TANs, CAF-mediated physical barriers, TIGIT/NECTIN2 checkpoint axis), constitutes the core mechanistic architecture underlying primary resistance and relapse.

**What are the implications of the main findings?**

- By framing neuroblastoma as a continuously evolving ecosystem under therapeutic pressure, this work provides a rational roadmap for next-generation combination strategies—including epigenetically re-sensitizing MES-state cells, temporally sequenced TME remodeling prior to immunotherapy, and dual-targeting CAR designs—moving beyond empirical one-size-fits-all approaches toward mechanism-guided precision intervention.
- The integrated multidimensional biomarker framework proposed herein (incorporating MES signature scores, TAM subset abundance, FcγRIIIa genotyping, and ctDNA-based dynamic monitoring) offers a clinically actionable strategy for early identification of non-responders, real-time detection of emerging resistance, and patient stratification in future trial designs.

**Abstract:**

Neuroblastoma is the most common extracranial solid tumor in children, and the prognosis for high-risk patients remains dismal. Immunotherapies, represented by anti-GD2 monoclonal antibodies and chimeric antigen receptor T cells (CAR-T), have significantly improved the survival of high-risk neuroblastoma patients and become part of standard therapy. However, their efficacy exhibits significant inter-individual heterogeneity, with some patients showing primary resistance or secondary relapse. This review aims to analyze the multi-faceted factors influencing the response to immunotherapy in neuroblastoma, including: (1) the inherent immunosuppressive properties of the tumor microenvironment, such as infiltration of myeloid-derived suppressor cells and tumor-associated macrophages, as well as checkpoint molecules and metabolic barriers; (2) tumor cell-intrinsic characteristics, such as low tumor mutational burden, *MYCN* amplification-associated downregulation of MHC-I, and heterogeneity of GD2 antigen expression; (3) host factors, such as systemic immune status and Fc receptor polymorphisms; and (4) treatment-related factors, such as combination strategies and the development of novel immunotherapeutic products. A deep understanding of these interrelated factors is crucial for developing predictive biomarkers, designing novel combination strategies and next-generation immunotherapies, and ultimately achieving precise immunotherapy for neuroblastoma.

## 1. Introduction

Neuroblastoma (NB) is the most common extracranial solid tumor in children, with 90% of cases occurring before the age of 5 years, accounting for 10% of pediatric cancer-related deaths [1,2]. This tumor originates from undifferentiated sympathetic neural crest cells and exhibits extremely high heterogeneity in its clinical course and biological behavior [3], ranging from spontaneous regression to widespread metastasis [4]. Based on the International Neuroblastoma Staging System and incorporating diagnostic age, histopathology, and key molecular features (such as ploidy, *MYCN* oncogene amplification, 11q aberration), patients are stratified into very-low-risk, low-risk, intermediate-risk, and high-risk groups to guide treatment [5]. While low- and intermediate-risk children often achieve good outcomes with surgery with or without low-intensity chemotherapy, high-risk neuroblastoma (HR-NB) (accounting for approximately 40–50% of new cases) constitutes a major challenge in pediatric oncology [6]. Historically, multi-modal intensive treatment for HR-NB, including high-dose induction chemotherapy, surgery, consolidation with autologous hematopoietic stem cell transplantation, radiotherapy, and maintenance therapy with 13-cis-retinoic acid, has improved 5-year survival to approximately 50%. However, nearly half of the patients still face relapse or progression, and the acute adverse effects and long-term sequelae of traditional intensive chemotherapy are concerning [7]. Therefore, developing more effective and tolerable novel treatment strategies is an urgent need to improve outcomes for children with HR-NB.

In this context, immunotherapy targeting the tumor-associated glycolipid antigen disialoganglioside GD2 has brought a revolutionary breakthrough to the treatment landscape of HR-NB. The pivotal phase III trial (COG ANBL0032) demonstrated that adding immunotherapy with the anti-GD2 monoclonal antibody (Dinutuximab) in combination with granulocyte-macrophage colony-stimulating factor and interleukin-2 during the maintenance phase after standard therapy significantly improved event-free survival and overall survival [8]. Studies show that incorporating anti-GD2 antibody therapy into standard care improved event-free survival in high-risk neuroblastoma by approximately 15%. Consequently, Dinutuximab combined with cytokines has become the international standard of care for frontline consolidation therapy in HR-NB [9]. Furthermore, chimeric antigen receptor T-cell therapies targeting GD2 and other targets (e.g., B7-H3) have shown encouraging anti-tumor activity in clinical trials for relapsed and refractory neuroblastoma [10,11,12].

However, clinical responses to immunotherapy show marked inter-individual heterogeneity. A significant proportion of patients exhibit primary resistance to GD2-targeted therapy or relapse after treatment [13]. Even the promising CAR-T therapy faces key challenges in neuroblastoma, such as limited tumor infiltration, insufficient persistence, and suppression by the tumor microenvironment [14]. Despite advances in multi-modal therapy, the long-term event-free survival rate for high-risk neuroblastoma patients remains suboptimal [15]. These clinical observations profoundly reveal that the sensitivity of neuroblastoma to immunotherapy is finely regulated by a complex network of multi-layered factors. Therefore, a systematic analysis of the key determinants of efficacy—from the inhibitory tumor microenvironment and intrinsic tumor cell properties to individual patient differences and therapeutic strategy design—holds significant clinical importance for overcoming current therapeutic bottlenecks, optimizing existing regimens, and developing next-generation immunotherapy strategies. This review will summarize recent research progress focusing on these four core aspects.

## 2. Intrinsic Immunosuppressive Characteristics of the Tumor Microenvironment

**Figure 1:**
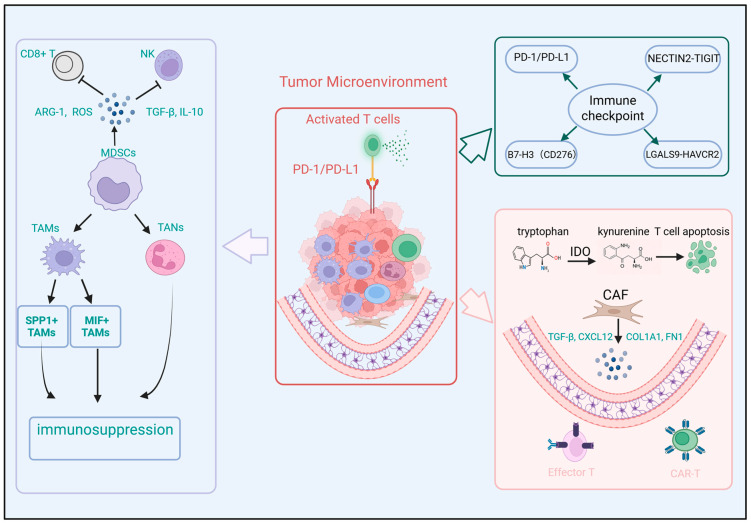
The intrinsic immunosuppressive properties of the tumor microenvironment. The immunosuppressive tumor microenvironment of neuroblastoma constitutes a multi-layered defense system comprised of immunosuppressive cells, immune checkpoint molecules, and physical metabolic barriers. MDSCs suppress the function of CD8+ T cells and NK cells through the release of cytokines such as ARG-1 and ROS, and also exert immunosuppressive effects via TAMs and TANs. In addition, neuroblastoma cells directly interfere with immune cell function through the upregulation of various immune checkpoint molecules, including PD-1/PD-L1. Furthermore, physical barriers established by CAFs and the deposition of kynurenine contribute to resistance against immune effector cells. MDSCs: Myeloid-derived suppressor cells; NK: natural killer; ARG-1: arginase-1; ROS: reactive oxygen species; TAMs: Tumor-associated macrophages; TANs: tumor-associated neutrophils; IDO: Indoleamine 2,3-dioxygenase; CAFs: cancer-associated fibroblasts.

Neuroblastoma is a typical “immunologically cold tumor”, characterized by scarce infiltration of effector immune cells (e.g., cytotoxic T lymphocytes) alongside a highly active network of immunosuppressive cells and molecules, collectively constructing a microenvironment conducive to tumor growth and immune evasion (Figure 1) [16]. This inhibitory immune microenvironment is a key intrinsic barrier limiting the efficacy of current immunotherapies such as anti-GD2 antibodies and CAR-T cells.

### 2.1. Infiltration and Function of Immunosuppressive Cells

Specific immune cell populations infiltrating the tumor microenvironment are the core executors of immunosuppression. Myeloid-derived suppressor cells (MDSCs) are significantly expanded in the peripheral blood and tumor tissues of neuroblastoma patients. These cells potently suppress the function of CD8+ T cells and natural killer (NK) cells by releasing arginase-1 (ARG-1), inducible nitric oxide synthase, reactive oxygen species (ROS), and inhibitory cytokines (e.g., TGF-β, IL-10), serving as a major driver of systemic immunosuppression [17,18]. Tumor-associated macrophages (TAMs) are abundant in neuroblastoma and predominantly polarized towards an M2-like phenotype with pro-tumor, pro-angiogenic, and immunosuppressive functions. M2 TAMs directly inhibit T-cell activation, promote regulatory T-cell function, and remodel the extracellular matrix by secreting mediators like TGF-β, IL-10, and VEGF, correlating with poor patient prognosis [19,20]. Single-cell atlases reveal significant heterogeneity and plasticity among TAMs. Studies have identified specific TAM subsets enriched in the NB TME, such as SPP1+ TAMs and MIF+ TAMs, which drive immunosuppression and stromal remodeling through distinct signaling axes [21]. SPP1+ TAMs promote angiogenesis and extracellular matrix (ECM) reorganization via the SPP1-ITGB1-STAT3 axis, while MIF+ TAMs mediate immunosuppressive interactions through the MIF-CD74/CD44/CXCR4 signaling network [22]. These TAMs act synergistically with tumor-associated neutrophils (TANs). TANs highly express CXCL8 and S100A8/A9, activating the CXCR2-MAPK/NF-κB pathway, inducing T-cell exhaustion and upregulating inhibitory molecules like ARG1 and PD-L1 [23]. Furthermore, intratumoral enrichment of regulatory T cells (Tregs) is also associated with an immunosuppressive state and poor prognosis. Tumor-infiltrating Tregs suppress the proliferation and function of effector T cells through various contact-dependent and -independent mechanisms (e.g., IL-2 consumption, CTLA-4 expression). High levels of Treg infiltration correlate with an immunosuppressive microenvironment and worse clinical outcomes in neuroblastoma [24].

### 2.2. Multi-Layered Immune Checkpoints and Inhibitory Signals

Beyond cellular suppression, neuroblastoma directly interferes with immune cell function by upregulating various immune checkpoint molecules. The classic PD-1/PD-L1 pathway is significantly associated with patient prognosis [25]. B7-H3 (CD276) is stably and highly expressed in the vast majority of neuroblastomas. Besides its expression level correlating with *MYCN* amplification status and high-risk disease, B7-H3 inhibits the activation and cytokine secretion of T cells and NK cells, acting as an important inhibitory immune checkpoint [26,27], and playing a key role in tumor cell proliferation, migration, and invasion [28]. Additionally, single-cell interaction analysis identified the NECTIN2-TIGIT axis as a crucial immunosuppressive pathway in NB, whose activation is closely associated with T-cell exhaustion phenotypes [29]. Spatial multi-omics analysis further revealed the role of vascular-immune interactions in maintaining the inhibitory environment, such as enriched LGALS9-HAVCR2 (TIM-3) interactions between endothelial cells and NK/T cells [30]. These molecules collectively constitute a multi-layered network of immunosuppressive signals. The mechanistic basis of B7-H3-mediated immunosuppression extends beyond simple checkpoint function. B7-H3 modulates multiple intracellular signal transduction pathways in tumor cells, including JAK/STAT, PI3K/Akt/mTOR, ERK, and NF-κB, thereby influencing cancer cell metabolism, promoting invasion, metastasis, and resistance to anticancer therapy [31]. Importantly, B7-H3 expression is regulated at the post-transcriptional level by microRNAs, particularly miR-29 family members, which are downregulated in neuroblastoma, leading to B7-H3 upregulation [32]. The clinical significance of B7-H3 is underscored by its correlation with advanced tumor stage and decreased overall survival, and notably, B7-H3 remains highly expressed even in GD2-negative tumors, positioning it as a complementary therapeutic target for patients experiencing anti-GD2 resistance [33]. The NECTIN2-TIGIT axis has emerged as a particularly promising target based on recent integrative single-cell RNA sequencing analyses of 24 neuroblastoma tumors (10 pre- and 14 post-chemotherapy, including 5 paired samples). This comprehensive study revealed that neuroblastomas are infiltrated by NK cells showing reduced cytotoxicity and T cells with dysfunctional profiles. Interaction analysis identified NECTIN2-TIGIT as a crucial immune checkpoint, and combined blockade of TIGIT and PD-L1 significantly reduced neuroblastoma growth in vivo, with complete responses achieved in a chemotherapy-resistant Th-ALKF1174L/*MYCN* syngeneic model [29,34]. These findings provide a strong rationale for incorporating TIGIT/PD-L1 combination blockade into immunotherapeutic strategies for high-risk neuroblastoma.

Beyond these well-characterized checkpoints, the neuroblastoma immune landscape is shaped by additional layers of regulation. TIM-3 (HAVCR2) interactions with LGALS9 on endothelial cells contribute to vascular-immune crosstalk that maintains the inhibitory microenvironment [30]. Furthermore, neuroblastoma exhibits features of an immunologically “cold” tumor, including low mutational burden, scant expression of neoepitopes, and loss of HLA Class I molecules, which collectively limit T cell recognition and contribute to the limited efficacy of single-agent checkpoint inhibitors [35,36]. This immune-cold phenotype, paradoxically combined with high expression of multiple checkpoints, necessitates combinatorial approaches that simultaneously target both tumor-intrinsic immunosuppressive pathways and the dysfunctional immune microenvironment. The translational relevance of these findings is reflected in the growing number of clinical trials targeting these checkpoints. Enoblituzumab, an anti-B7-H3 monoclonal antibody, has completed Phase I testing in children with B7-H3-expressing solid tumors (NCT02982941). Multiple B7-H3 CAR-T cell trials are currently recruiting for neuroblastoma patients, with early-phase studies evaluating safety and efficacy (NCT04691713, NCT04432649, NCT04897321) [33]. Radioimmunotherapy approaches using 131I-Omburtamab are also in active clinical development for neuroblastoma with CNS/leptomeningeal metastases (NCT03275402). These ongoing trials, together with the strong preclinical rationale for TIGIT/PD-L1 combination blockade, underscore the potential of multi-layered checkpoint targeting to improve outcomes in high-risk neuroblastoma. Clinical evidence for checkpoint inhibitor combinations in neuroblastoma is emerging. A pilot study of two patients with refractory neuroblastoma treated with nivolumab (anti-PD-1) combined with dinutuximab beta demonstrated promising activity: one patient achieved complete remission lasting >6 months, and the other achieved very good partial remission with substantial regression of skeletal lesions. Tolerance to the combination was excellent [37].

### 2.3. Establishment of Physical and Metabolic Barriers

The tumor microenvironment also resists immune effector cells by establishing physical and metabolic barriers. Physically, neuroblastoma tissues often exhibit excessive deposition of extracellular matrix (particularly collagen and fibronectin) and a disorganized, dysfunctional vasculature. This dense stromal network forms a physical barrier, severely hindering the effective infiltration and cytotoxic activity of effector T cells and CAR-T cells into the tumor parenchyma [38,39]. Cancer-associated fibroblasts (CAFs) are the primary architects of this physical barrier. Single-cell analysis has identified immunosuppressive CAF subsets that secrete large amounts of TGF-β, CXCL12, COL1A1, and FN1, forming a collagen-rich dense physical structure that excludes effector T cells from the tumor parenchyma, creating an “immune-excluded” zone [40].

Notably, the physical barrier established by cancer-associated fibroblasts (CAFs) and a collagen-rich stroma is not a static structure; rather, it exhibits significant spatiotemporal heterogeneity across different anatomical sites and disease stages. In the bone marrow, the most common metastatic site for neuroblastoma, the microenvironment displays unique properties: bone marrow-derived mesenchymal stem cells (BM-MSCs) can be converted into α-Smooth Muscle Actin (αSMA)-positive CAFs under the influence of tumor cells, thereby contributing to the construction of this physical barrier [41]. More importantly, clinical sample analyses have confirmed that high expression of COL11A1, a key collagen component of the physical barrier, in CAFs is significantly associated with tumor stage, tumor status, relapse, and poor overall survival [42]. Recent single-cell studies have further revealed an enrichment of mesenchymal phenotypes in chemotherapy-resistant cells and dynamic phenotypic switching in relapsed tumors [43], suggesting that therapeutic pressure itself remodels tumor cell states and their physical interactions with the microenvironment. Furthermore, tumor cells can actively sense and respond to the biomechanical properties of the stroma via collagen receptors such as Discoidin Domain Receptor Tyrosine Kinase 2 (DDR2); high DDR2 expression is correlated with poor patient survival [44]. A comprehensive review by Horwacik clearly indicates that the composition, organization, and signaling networks of the extracellular matrix (ECM) are significantly remodeled during tumor progression and metastasis, with CAFs, as depositors of ECM structural components, playing a central role in shaping this physical barrier [45]. Similarly, a review by Jahangiri establishes the association between relapse and the ECM as a core research focus, emphasizing that the tumor microenvironment (TME) can influence disease progression and patient prognosis [46]. Through single-cell sequencing of paired pre- and post-chemotherapy samples from patients with high-risk neuroblastoma, researchers have found a significant increase in fibroblasts and macrophages within the TME following chemotherapy. These remodeled microenvironmental cells promote tumor cell proliferation and drug resistance by secreting molecules such as Heparin-Binding EGF-like Growth Factor (HB-EGF) [22]. This indicates that therapeutic pressure itself can “select for” and shape a physical microenvironment with enhanced pro-tumorigenic and immunosuppressive properties, leading to a denser physical barrier surrounding the tumor at relapse. In summary, these findings collectively suggest that the physical barrier is dynamically remodeled and reinforced during metastasis and relapse, constituting a critical mechanism of resistance to immunotherapy.

Metabolically, rapidly growing tumors often cause local hypoxia. This hypoxic state induces the expression of factors like HIF-1α, driving angiogenesis, epithelial-mesenchymal transition, and promoting the expression of various immunosuppressive genes (e.g., PD-L1), while simultaneously inhibiting effector T-cell function [47]. Regarding nutrient competition and metabolites, neuroblastoma cells and immunosuppressive cells in the microenvironment (e.g., MDSCs, M2 macrophages) highly express indoleamine 2,3-dioxygenase (IDO). This enzyme catalyzes the breakdown of tryptophan to kynurenine, leading to local tryptophan depletion. The accumulation of kynurenine has direct cytotoxic and anti-proliferative effects on T cells, inducing T-cell dysfunction and apoptosis [48].

IDO/tryptophan metabolism represents only one of the metabolic pathways involved in tumor-mediated immune tolerance. Indeed, metabolites associated with aerobic glycolysis, as well as the metabolism of adenosine, arginine, and prostaglandins, all participate in cancer-mediated immune tolerance, collectively constituting the “next-generation metabolic immune checkpoints” [49]. Recent studies have confirmed that neuroblastoma cells promote tumor progression through the kynurenine-AHR pathway, and high expression of genes in this pathway is significantly associated with poor prognosis [50]. Beyond the aforementioned pathways, the neuroblastoma tumor microenvironment (TME) poses multiple challenges to CAR-T cell function through broader metabolic remodeling. Specifically, high-risk neuroblastoma driven by *MYCN* amplification exhibits metabolic reprogramming characteristics, influencing T cell function through the dual mechanisms of metabolic competition and metabolic antagonism [51]. At the level of metabolic competition, tumor cells extensively consume glucose through aerobic glycolysis (the Warburg effect), leading to glucose depletion within the TME [52]. The effector function of CAR-T cells is highly dependent on glycolysis; however, glucose deficiency is prevalent in the tumor microenvironment [53]. This forces infiltrating T cells into a critical competition with tumor cells for limited glucose—a competition that directly impairs T cell function and promotes the formation of exhaustion phenotypes. At the level of metabolic antagonism, the substantial lactate exported from tumor cells due to high-rate glycolysis leads to acidification of the TME. Lactate directly suppresses the function of CD8+ effector T cells by inhibiting pyruvate carboxylase activity and the expression of Nuclear Factor of Activated T cells (NFAT), while simultaneously promoting the infiltration of myeloid-derived suppressor cells (MDSCs) and M2 macrophage polarization [51]. Notably, the metabolic adaptability of CAR-T cells is closely related to their design: the 4-1BB costimulatory domain promotes oxidative phosphorylation and the formation of central memory T cells, whereas CAR-T cells dominated by the CD28 costimulatory domain primarily rely on glycolysis and differentiate into effector memory T cells. This suggests that metabolic adaptability can be improved through optimized design [54]. Furthermore, studies reveal that mesenchymal (MES)-state NB cells exhibit unique metabolic reprogramming, relying on oxidative phosphorylation and fatty acid oxidation, a metabolic state associated with therapy resistance [55]. In response to these multifaceted metabolic challenges, “nutrient gene therapy” strategies, such as overexpressing GLUT1 to enhance glucose uptake, hold promise for augmenting the metabolic competitive capacity of CAR-T cells against tumor cells, thereby overcoming T cell exhaustion [52].

In summary, the neuroblastoma tumor microenvironment is a complex defense system jointly constructed by inhibitory cells, inhibitory signals, and physical-metabolic barriers. A profound understanding and targeting of these immunosuppressive mechanisms are essential for breaking tumor immune evasion and unlocking the full potential of immunotherapy.

## 3. Intrinsic Characteristics of Tumor Cells

**Figure 2:**
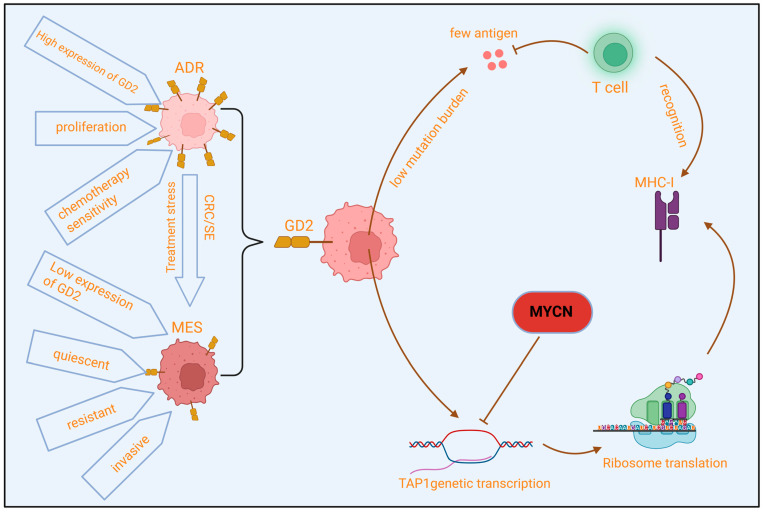
Intrinsic Characteristics of Tumor Cells. Neuroblastoma cells predominantly exist in two interconvertible states, ADR and MES. Therapeutic pressure induces a phenotypic switch from the ADR to the MES state, leading to increased aggressiveness and treatment resistance. The relatively stable genome of neuroblastoma results in a limited number of neoantigens generated by mutations, thereby lacking high-affinity targets recognizable by T cells and exhibiting weak inherent immunogenicity. Furthermore, amplification of the *MYCN* oncogene directly suppresses the transcription of MHC-I-related genes, impairing the function of antigen processing and presentation pathways, and rendering tumor cells invisible to T cell-mediated immune surveillance. ADR: adrenergic; MES: mesenchymal; CRC: core transcription regulatory circuitry; SE: super enhancer; MHC-I: major histocompatibility complex class I.

The intrinsic properties of tumor cells are fundamental in determining their susceptibility to immune recognition and elimination. Neuroblastoma cells employ various intrinsic mechanisms to actively diminish their immunovisibility and evolve strategies to counteract targeted therapies (Figure 2).

### 3.1. Low Immunogenicity, Antigen Presentation Defects, and Lineage Plasticity

Compared to “hot tumors” with high mutational burden like melanoma or lung cancer, neuroblastoma has a relatively stable genome and a low somatic mutation rate [56]. This directly results in a limited number of neoantigens generated from mutations, leading to an inherently weak immunogenicity due to the lack of tumor-specific, high-affinity T-cell targets [57]. Antigen presentation is central to T-cell immunity. In neuroblastoma, especially in high-risk subtypes, the surface expression of major histocompatibility complex class I (MHC-I) molecules on tumor cells is frequently downregulated or even lost [58]. A key driver of this defect is the amplification of the *MYCN* oncogene. *MYCN* is a critical driver gene and high-risk marker in neuroblastoma, and studies have confirmed that it acts as a transcription factor to directly repress the transcription of MHC-I pathway-related genes (e.g., *TAP1*, β2-microglobulin), impairing antigen processing and presentation [59]. This prevents effective presentation of tumor antigens to CD8^+^ cytotoxic T cells, making tumor cells invisible to T-cell surveillance—a crucial immune evasion mechanism [60]. Additionally, single-cell studies have unveiled the dynamic lineage plasticity of NB tumor cells. Tumor cells primarily exist in two interconvertible states: adrenergic (ADR) and mesenchymal (MES) [61]. ADR-state cells express neuronal markers (e.g., *TH*, *PHOX2B*), are proliferative, and are initially sensitive to chemotherapy. In contrast, MES-state cells express stromal and mesenchymal markers (e.g., *VIM*, *PRRX1*), are more invasive, quiescent, and therapy-resistant [62,63]. This ADR-MES plasticity is driven by the reprogramming of the core transcription regulatory circuitry (CRC) and super-enhancers (SE). Upregulation of key transcription factors (e.g., PRRX1), loss of chromatin remodeler components (e.g., ARID1A), or activation of the NOTCH3 signaling pathway can drive the transition from an ADR to a MES transcriptional landscape, thereby promoting cell identity switch and drug resistance [64,65,66]. Therapeutic pressure (e.g., chemotherapy) selects for and enriches MES-state cells, forming an important cellular basis for acquired resistance [67].

### 3.2. Heterogeneity, Dynamic Loss, and Regulation of Target Antigen Expression

The expression of GD2, the primary target for immunotherapy, is profoundly influenced by tumor heterogeneity and lineage plasticity. Intra-tumoral heterogeneity exists with subpopulations expressing GD2 at varying levels, providing a basis for antigen escape. Persistent anti-GD2 therapeutic pressure may also lead to downregulation or loss of the target epitope [68]. When GD2-targeted immunotherapy eliminates high-expressing clones, low-expressing or non-expressing clones gain a selective advantage, proliferate, and ultimately lead to disease relapse [69]. More importantly, the transition to a MES state actively leads to downregulation of GD2 expression. The mechanism involves the silencing of key GD2-synthesis enzyme genes like *ST8SIA1* within the MES-associated epigenetic program, directly explaining why MES-type NB cells are less sensitive to GD2-based antibody or CAR-T therapy [70]. Through low immunogenicity, antigen presentation defects, and heterogeneous and unstable target antigen expression, tumor cells establish multiple intrinsic lines of defense. These characteristics not only explain why neuroblastoma is difficult for the innate immune system to clear but also directly challenge the efficacy and durability of current targeted immunotherapies, highlighting the necessity of developing multi-targeted or antigen-independent novel strategies.

## 4. Host Factors

**Figure 3:**
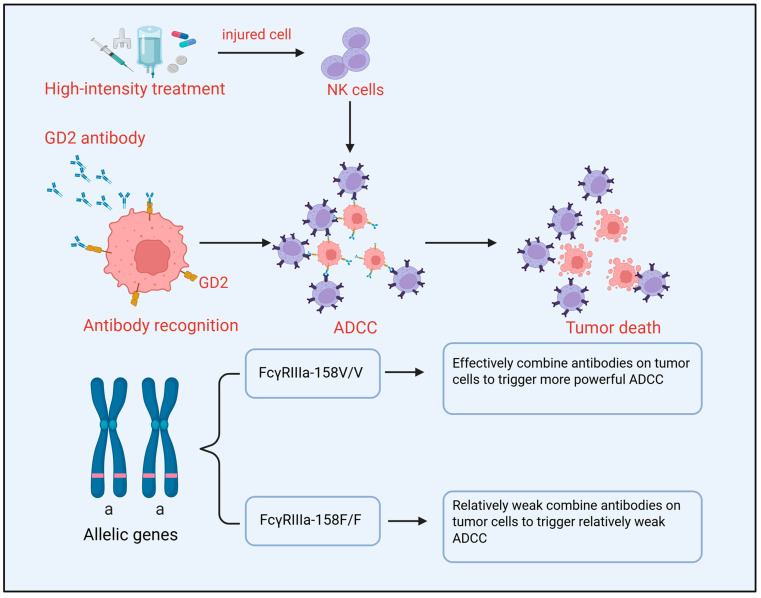
Individual patient differences directly influence the tolerability and effectiveness of immunotherapy. GD2 antibody primarily exert their effects through ADCC, an effector function that relies on the abundance and activity of NK cells in vivo. High-intensity chemotherapy induces damage to NK cells, directly compromising the ADCC response and leading to poor clinical outcomes. In addition, Fcγ receptor gene polymorphisms also influence the efficacy of ADCC. ADCC: Antibody-dependent cell-mediated cytotoxicity; NK: natural killer.

Individual patient differences directly influence the tolerability and effectiveness of immunotherapy. The patient’s genetic background, systemic immune status, and pharmacokinetic characteristics constitute the “host” context for immunotherapy response, profoundly affecting drug efficacy and toxicity (Figure 3).

### 4.1. Systemic Immune Status

The standard treatment paradigm for high-risk neuroblastoma, including intensive induction chemotherapy, autologous hematopoietic stem cell transplantation, radiotherapy, and subsequent immunotherapy maintenance, aims to eradicate the tumor. However, these treatments, particularly myeloablative chemotherapy and transplantation, induce severe and long-lasting lymphocytopenia and impair immune system reconstitution capacity. This may weaken antibody-dependent cell-mediated cytotoxicity (ADCC), which relies on host effector cells like NK cells [71]. This immunosuppressed state poses a challenge for immunotherapies dependent on functional host effector cells. For example, anti-GD2 monoclonal antibodies (e.g., Dinutuximab) primarily function via ADCC, the efficacy of which is highly dependent on the abundance and function of cytotoxic NK cells and macrophages in the host [72]. In the context of incomplete immune reconstitution, insufficient numbers or impaired function of effector cells can directly diminish the ADCC effect, leading to suboptimal clinical response. Therefore, optimizing the timing of immunotherapy (e.g., during the consolidation and maintenance phase when the immune system has partially recovered) or combining it with immune modulators like granulocyte-macrophage colony-stimulating factor (GM-CSF) or interleukin-2 (IL-2) to activate and expand effector cells has become part of the clinical standard.

### 4.2. Fcγ Receptor Gene Polymorphisms

Beyond the number and functional state of effector cells, their killing capacity is regulated by genetic factors. A key mediator of ADCC is the Fcγ receptor IIIa (FcγRIIIa, CD16a) expressed on effector cells (primarily NK cells, also monocytes/macrophages). The human *FCGR3A* gene harbors functional single-nucleotide polymorphisms, most notably at amino acid position 158, resulting in a valine (V) or phenylalanine (F) variant (V158F) [73]. This variant significantly affects receptor affinity for the antibody Fc region: the FcγRIIIa-158V allele (especially the *v*/*v* homozygous genotype) encodes a receptor with much higher affinity for human IgG1 (e.g., Dinutuximab) than the receptor encoded by the 158F allele [74]. This affinity difference has direct clinical implications. Multiple retrospective and prospective studies have confirmed that neuroblastoma patients carrying the high-affinity FcγRIIIa-158 *v*/*v* genotype exhibit significantly superior clinical outcomes following Dinutuximab-based immunotherapy, including higher event-free survival and overall survival rates [75]. The mechanism lies in the more efficient binding of antibodies on tumor cells by effector cells in *v*/*v* genotype patients, triggering more potent ADCC and more thorough clearance of minimal residual disease. Conversely, patients carrying the low-affinity F allele (especially F/F homozygotes) may require higher antibody exposure or additional immune stimulation to achieve comparable efficacy. Pandey et al. demonstrated through ADCC inhibition assays that the anti-GD2 antibody-mediated ADCC function of NK cells carrying the low-affinity FcγRIIIa-F/F genotype can be significantly inhibited by endogenous immunoglobulins, whereas *v/v* homozygotes possess resistance to such inhibition [76]. This finding suggests that patients with the F/F genotype may require differentiated treatment strategies to achieve clinical benefits comparable to those observed in patients with the *v*/*v* genotype. Therefore, *FCGR3A* genotyping is considered a potential biomarker for predicting the efficacy of anti-GD2 monoclonal antibodies, providing a theoretical basis for future personalized treatment strategies (e.g., adjusting dosage or combination regimens based on genotype) [77].

Currently, patient stratification based on FCGR genotypes has entered the clinical trial stage—the COG ANBL1221 study has systematically evaluated the impact of single-nucleotide polymorphisms in FCGR3A and other genes on the efficacy of chemoimmunotherapy regimens [78]. Interventional strategies for patients with the F/F genotype can be explored from multiple dimensions: First, the enhancement of adjuvant cytokine support. In addition to its role as a standard adjunctive agent, GM-CSF can partially compensate for NK cell ADCC deficiencies by augmenting the antibody-dependent cellular phagocytosis (ADCP) function of myeloid effector cells (macrophages and monocytes). Studies on engineered Fc variants by Kang et al. demonstrated that GM-CSF-differentiated macrophages, despite exhibiting low FcγRIIIa expression levels, can still mediate potent ADCP through selective activation—a finding that provides a mechanistic basis for GM-CSF intensification strategies [79]. Second, the incorporation of NK cell activation strategies. Research by Lode et al. revealed that KIR haplotypes are also associated with clinical outcomes: patients carrying the stimulatory KIR haplotype (2DS2+) exhibited significantly superior progression-free survival and overall survival compared to those with inhibitory KIR haplotypes or KIR/KIR-ligand matching [80]. Further confirmation came from Siebert et al. in Oncoimmunology, demonstrating that the presence of activating KIR 2DS2 significantly influences antibody-dependent cellular cytotoxicity levels and patient survival, with the most pronounced benefit observed in 2DS2-positive patients who also carried high-affinity FCGR2A and FCGR3A genotypes [81]. Analyses by Erbe et al. of the Children’s Oncology Group (COG) ANBL0032 phase III trial similarly indicated that KIR/KIR-ligand genotypes can influence clinical outcomes of dinutuximab-based immunotherapy, with KIR/KIR-ligand genotypes deriving more benefit from immunotherapy [78]. These findings collectively suggest that incorporating KIR-directed NK cell activation strategies (e.g., enhancing NK cell function via IL-2) may hold significant clinical value for patients with the F/F genotype. Third, the development of Fc-engineered antibodies. Enhancing antibody binding affinity to FcγRIIIa through afucosylation modification represents a well-established strategy for improving ADCC. The mechanistic basis for this enhancement lies in the removal of core fucose from the Fc N-glycan, which eliminates steric hindrance between the antibody Fc and FcγRIIIa, leading to enhanced activation of proximal signaling components, cytoskeletal rearrangement, and more efficient serial killing by NK cells [82]. Beyond simple afucosylation, the specific location of galactosylation on Fc N-glycans has emerged as a critical determinant of FcγRIIIa binding affinity. Recent studies demonstrate that galactosylation of the α6 antenna, but not the α3 antenna, of afucosylated biantennary glycans significantly increases FcγRIIIa binding and ADCC activity, highlighting the importance of precise glycan pairing and isomer-specific glycosylation [83,84].

The combination of glycoengineering with targeted Fc point mutations represents an even more powerful strategy. Studies have shown that combining afucosylation with specific Fc point mutations (such as S239D/I332E) greatly increases antibody affinity for and retention on FcγRIIIa, enhancing immune effector activity at doses suboptimal for wild-type antibodies and recruiting FcγRIIIa-expressing effector cells into tumors [85]. The Fc3aV engineered Fc variant developed by Kang et al., which selectively binds FcγRIIIaV158, demonstrates the feasibility of antibody engineering to enhance binding to specific FcγR subtypes [79]. An innovative approach to Fc engineering involves switching antibody isotypes entirely. IgA3.0 ch14.18, an engineered IgA antibody targeting GD2, engages the Fcα receptor (CD89) on neutrophils and macrophages rather than FcγRIIIa on NK cells [86]. This format offers two potential advantages for F/F genotype patients: (1) it bypasses FcγRIIIa-mediated ADCC entirely, potentially overcoming genotype-related deficiencies, and (2) it may reduce neuropathic pain associated with GD2-targeted therapy by avoiding binding to GD2-expressing sensory neurons [85]. These diverse Fc engineering strategies—glycoengineering, point mutations, and isotype switching—collectively hold promise for directly overcoming receptor-level affinity deficiencies and improving outcomes for patients with the F/F genotype. In summary, patient stratification based on FCGR3A genotype has demonstrated clinical feasibility, and future prospective studies should focus on validating optimized treatment strategies tailored for patients with the F/F genotype.

### 4.3. Other Host Genetic and Pharmacokinetic Factors

Beyond FcγRIIIa, other host factors that may influence the pharmacokinetics, pharmacodynamics, or toxicity of immunotherapies are gaining attention. These include factors affecting antibody clearance, genetic susceptibility loci related to adverse effects like cytokine release syndrome or pain, all of which may contribute to inter-patient variability in efficacy and toxicity [87,88,89]. With the advancement of precision medicine, a deeper understanding of these host factors will help optimize the therapeutic window, ensuring optimal efficacy while managing adverse effects.

In summary, while host factors do not directly alter tumor biology, they are crucial determinants of whether immunotherapy can succeed in an individual patient. Treatment-induced immune exhaustion, key Fcγ receptor polymorphisms, and other pharmacogenetic characteristics collectively shape immunotherapy outcomes. In the future, comprehensively assessing a patient’s immune reconstitution status and key genetic background when formulating treatment strategies holds promise for advancing neuroblastoma immunotherapy towards a more precise, personalized model.

## 5. Treatment-Related Factors

**Figure 4:**
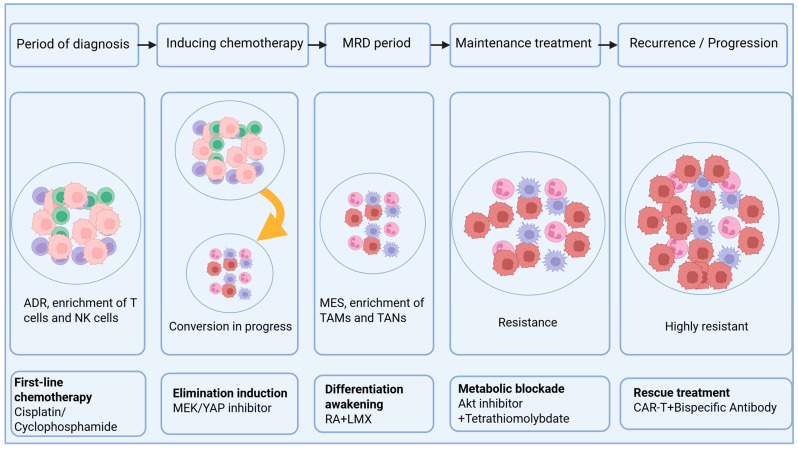
Temporally Sequenced Therapeutic Roadmap Targeting Neuroblastoma Cell State Transitions. The timeline (top) depicts five sequential treatment phases: Period of Diagnosis, Inducing Chemotherapy, MRD Period, Maintenance Treatment, and Recurrence/Progression. ADR cells are enriched with T cells and NK cells, while MES cells are enriched with TAMs and TANs. MES cells progressively enriched under therapeutic pressure, culminating in highly resistant populations at recurrence. Pharmacological interventions are positioned at their optimal timing of administration: First-line chemotherapy (Cisplatin/Cyclophosphamide): Administered during inducing chemotherapy to debulk the initial tumor burden. Elimination induction (MEK/YAP inhibitors): Targeted during the MRD window to eliminate persister cells by blocking survival signals. Differentiation awakening (RA + LMX): Combined retinoic acid and GART inhibitor to induce differentiation of quiescent MES cells. Metabolic blockade (Akt inhibitor + Tetrathiomolybdate): Applied during maintenance treatment to disrupt metabolic plasticity by simultaneously inhibiting glycolysis and oxidative phosphorylation. Rescue treatment (CAR-T + Bispecific Antibody): Reserved for recurrent/progressive disease to overcome multi-drug resistance. ADR: adrenergic; MES: mesenchymal; NK: natural killer; TAMs: Tumor-associated macrophages; TANs: tumor-associated neutrophils; RA: retinoic acid.

Treatment strategy and the tumor’s adaptive response jointly determine the final therapeutic outcome (Figure 4).

### 5.1. Therapy-Induced Remodeling of the Tumor Microenvironment and Cell State

During treatment, the neuroblastoma (NB) ecosystem undergoes profound remodeling that extends far beyond simple cytotoxic tumor reduction. Induction chemotherapy not only reduces tumor burden but also actively reprograms the cellular and epigenetic landscape, driving lineage conversion through multiple interconnected mechanisms. Studies have demonstrated that conventional chemotherapeutic agents (e.g., cisplatin, doxorubicin) can induce tumor cell transition from the proliferative adrenergic (ADR) state toward the invasive, quiescent mesenchymal (MES) state. This transition is driven by the upregulation of transcription factors such as PRRX1, alongside extensive remodeling of super-enhancer landscapes [65,90]. Recent mechanistic insights have further elucidated that this phenotypic switching is orchestrated by chromatin remodeling complexes—specifically, Xu et al. demonstrated that SWI/SNF ATPases (SMARCA2/4) are essential for establishing a MES gene-permissive chromatin state in ADR-type NB, and targeting these ATPases with dual degraders effectively inhibits cellular plasticity and prevents chemotherapy resistance [66].

The clinical significance of MES-state enrichment is underscored by its direct contribution to therapeutic resistance. MES-state cells exhibit marked chemoresistance and are selectively enriched under therapeutic pressure, constituting a reservoir of minimal residual disease (MRD) and serving as a critical source of subsequent relapse [91]. Uemura et al. have emphasized that MRD is composed of drug-resistant tumor cells dynamically presenting as cancer stem cells (CSCs) in residual tumors, circulating tumor cells (CTCs) in peripheral blood, and disseminated tumor cells (DTCs) in bone marrow and other metastatic sites, with epithelial-mesenchymal transition (EMT) programs serving as a key mechanism for acquiring MRD phenotypes [92]. Concurrently, the immune microenvironment evolves toward a profoundly immunosuppressive state through therapy-induced remodeling. A landmark longitudinal single-cell multiomic study by Liu et al., which profiled 22 high-risk NB patients before and after induction chemotherapy using paired snRNA-seq, snATAC-seq, and whole-genome sequencing, revealed profound shifts in immune composition [22]. Macrophages significantly expanded after therapy, polarizing toward pro-angiogenic, immunosuppressive, and metabolic phenotypes characterized by the expansion of SPP1+ tumor-associated macrophages (TAMs) and tumor-associated neutrophils (TANs). This was accompanied by the exhaustion of effector T cells and NK cells. Critically, this study identified a paracrine HB-EGF–ERBB4 signaling axis between macrophages and neoplastic subsets that promotes tumor growth through ERK pathway induction, providing a mechanistic link between therapy-induced TME remodeling and tumor progression [22]. This immunosuppressive and stroma-remodeled microenvironment becomes even more pronounced at relapse or metastatic stages, with fibroblasts and other stromal components further contributing to the physical barrier that impedes immune infiltration [93]. These coordinated changes—tumor cell state switching, MRD establishment, and immune microenvironment remodeling—collectively create a self-reinforcing ecosystem that drives therapeutic resistance and necessitates combinatorial strategies targeting both tumor-intrinsic plasticity and extrinsic immunosuppressive networks.

### 5.2. Multi-Layered Mechanisms of Resistance

Advances in research technologies, including single-cell sequencing, provide new avenues for further elucidating resistance mechanisms [94]. The core mechanisms driving treatment resistance can be conceptualized as a dynamic, multi-layered adaptive network. Within this network, the genetic and structural foundation of tumor cells is first reshaped; for instance, heterogeneity of extrachromosomal DNA can drive *MYCN* expression fluctuations and cell state transitions, providing the material basis for broad phenotypic plasticity [95]. Subsequently, epigenetic and core regulatory circuitry reprogramming is triggered, with the adrenergic-to-mesenchymal (ADR-MES) lineage switch identified as a central epigenetic mechanism driving resistance [64,65,66]. Beyond altering their own state, tumor cells actively modify their interactions with the environment. On one hand, they directly evade immune attack by downregulating therapeutic targets (e.g., GD2). On the other hand, they maintain survival networks through target loss and bypass activation. For example, heparin-binding epidermal growth factor secreted by tumor-associated macrophages can bypass therapeutic pressure by activating the ERBB4 receptor on tumor cells, sustaining key survival pathways like MAPK/PI3K [22]. Some tumor cells adopt a more “stealthy” survival strategy by significantly reducing transcriptional activity and entering a slow-cycling or quiescent state to tolerate therapy, forming so-called persister cells. These cells exhibit unique epigenetic and metabolic signatures and are a significant source of tumor relapse [96,97]. Simultaneously, tumor cells also exhibit defects in immune and inflammatory sensing, often lacking cGAS-STING-mediated innate immune sensing, limiting their self-immunogenicity. Interestingly, however, cells in the MES state show enhanced TLR3-mediated double-stranded RNA sensing capability, which unexpectedly provides a potential entry point for targeting these refractory cells with specific immune agonists [98]. Finally, metabolic adaptation to therapeutic pressure constitutes the ultimate line of defense. MES and persister cells generally exhibit enhanced oxidative phosphorylation, fatty acid oxidation, and antioxidant capacity (e.g., NRF2 pathway activation). Crucially, they can establish an autocrine loop by endogenously synthesizing all-trans retinoic acid (RA) via the ALDH1A1/3-mediated pathway, thereby resisting differentiation therapy with exogenous RA (e.g., 13-cis-retinoic acid), a key mechanism of resistance to current differentiation therapies [99,100].

For the eradication of persister cells during the minimal residual disease (MRD) window, a temporally sequenced combination therapeutic strategy can be designed. Recent research by Grossmann et al. has revealed that residual persister cells following chemotherapy gain a survival advantage by inhibiting *MYCN* activity and activating the NFκB signaling pathway [101]. Based on this mechanistic insight, we propose a three-stage interventional roadmap: The first stage (elimination induction) aims to target the survival signals of persister cells by employing the MEK inhibitor trametinib in combination with the YAP inhibitor CA3. Tsuji et al. have demonstrated that this combination regimen significantly prolongs progression-free survival in an MRD mouse model, with complete tumor clearance achieved in a subset of mice [102]. The second stage (differentiation awakening) focuses on the early MRD phase, combining retinoic acid (RA) with the purine metabolism inhibitor LMX. Jiang et al. discovered that LMX potently induces differentiation of *MYCN*-amplified neuroblastoma cells [103], exhibiting potential synergistic effects with RA and offering a strategy to awaken quiescent persister cells by driving them toward a more differentiation-prone and eliminable state. The third stage (metabolic blockade) targets persister cells that may escape the aforementioned interventions by combining an Akt inhibitor with the oxidative phosphorylation inhibitor tetrathiomolybdate, aiming to disrupt their metabolic plasticity and thereby cut off the energy supply routes of drug-resistant cells [104]. Building upon these three sequential stages, combination with anti-GD2 immunotherapy can be further incorporated to consolidate MRD clearance. Clinical data from Gorostegui et al. demonstrate that the addition of anti-GD2 immunotherapy during the MRD phase reduces the risk of relapse by 80% [105]. This temporally sequenced strategy, through multidimensional and multi-stage sequential interventions, holds promise for achieving complete eradication of persister cells and provides a novel therapeutic paradigm for preventing relapse in high-risk neuroblastoma.

### 5.3. New Insights for Combination Strategies

The aforementioned multi-layered resistance mechanisms provide direction for rationally designed combination therapies. Given that immunotherapy shows superior efficacy in the minimal residual disease state compared to bulky tumor burden, combination therapy is an inevitable direction to enhance efficacy.

First, immuno-combination strategies involve pairing anti-GD2 therapy with immune checkpoint inhibitors (e.g., antibodies targeting TIGIT, PD-L1) to reverse T-cell exhaustion and unleash a more robust anti-tumor immune response [106]. Second, interventions targeting the tumor microenvironment are key. Examples include using MIF inhibitors or CSF-1R inhibitors to target tumor-associated macrophages, aiming to remodel the immunosuppressive microenvironment and clear obstacles for immune cell (including CAR-T) function [107]. Simultaneously, addressing tumor cell-intrinsic resistance through epigenetic modulation (e.g., using EZH2 or BET inhibitors) can reverse the therapy-resistant mesenchymal state and upregulate target antigen GD2 expression, thereby overcoming antigen loss and restoring sensitivity to targeted therapy [70]. At the metabolic level, targeting the unique metabolic pathways on which resistant cells depend, such as key fatty acid oxidation enzymes like CPT1A, directly attacks their metabolic vulnerability [108]. For differentiation therapy resistance, strategies are needed to overcome endogenous RA tolerance, including targeting the endogenous RA synthesis pathway and combining it with exogenous RA or novel differentiating agents. Finally, optimization of the therapeutic products themselves is crucial. These diverse strategies, targeting both tumor-intrinsic and microenvironmental resistance mechanisms, provide a rich pipeline for rational combination therapy design in high-risk neuroblastoma.

### 5.4. CAR-T Cell Therapy: Optimization Strategies and Clinical Translation in Neuroblastoma

Chimeric antigen receptor (CAR)-T cell therapy has revolutionized the treatment of hematologic malignancies; however, its efficacy in solid tumors, including neuroblastoma, remains limited [109]. Early-phase clinical trials of GD2-targeted CAR-T cells have demonstrated safety and feasibility, yet clinical responses have been modest [110]. A recent systematic review and meta-analysis encompassing 8 studies with 146 neuroblastoma patients reported a pooled complete response rate of 39.57% and partial response rate of 15.83%, with hematologic toxicities being the most common adverse events [110]. These findings underscore the need for continued optimization of CAR-T cell products to enhance their anti-tumor activity in the neuroblastoma microenvironment.

CAR Design and Costimulatory Domains: The choice of costimulatory domains profoundly influences CAR-T cell function and persistence. Quintarelli et al. demonstrated that third-generation CARs targeting GD2 incorporating CD28.4-1BB costimulatory domains are associated with improved anti-tumor efficacy compared to those incorporating CD28.OX40 domains [111]. The inclusion of 4-1BB signaling resulted in significant amelioration of several CAR T-cell characteristics, including: (1) reduced T-cell exhaustion, (2) lower basal T-cell activation, (3) enhanced in vivo tumor control, and (4) improved T-cell persistence [111].

Dual-Targeting Strategies to Overcome Antigen Heterogeneity: Antigen escape remains a major challenge for single-target CAR-T approaches. Given that neuroblastoma cells exhibit heterogeneous expression of GD2, and GD2-negative clones can be selected under therapeutic pressure, dual-targeting strategies have emerged as a promising solution. Studies have demonstrated that dual-targeting CAR-T cells recognizing both GD2 and B7-H3—another immune checkpoint molecule highly expressed in neuroblastoma—can prevent antigen escape and provide sustained anti-tumor activity. A SynNotch-gated CAR-T system, where expression of the B7-H3 CAR is conditionally activated upon recognition of GD2, has shown remarkable specificity and efficacy in metastatic neuroblastoma xenograft models, with improved metabolic fitness and reduced exhaustion profiles compared to conventional CAR-T cells [112]. This gated approach may also mitigate on-target off-tumor toxicity, as CAR activity is restricted to tumor sites expressing both antigens.

Armored CAR-T Cells and Combination Strategies: To overcome the immunosuppressive tumor microenvironment, fourth- and fifth-generation “armored” CAR-T cells are being engineered to secrete immunomodulatory cytokines. Preclinical studies have shown that CAR-T cells engineered to secrete IL-15 or IL-7 exhibit enhanced persistence and function within the inhibitory TME [109]. The combination of CAR-T cells with immune checkpoint inhibitors represents another promising strategy. Toews et al. demonstrated that co-culturing neuroblastoma cell lines with L1CAM-CAR T cells upregulates PD-L1 expression on tumor cells and PD-1 on CAR-T cells, confirming adaptive immune resistance [113]. The addition of nivolumab (anti-PD-1) enhanced CAR-T cell-directed killing, with efficacy dependent on the strength of PD-1/PD-L1 axis expression in the CAR-T cells themselves. Notably, checkpoint inhibitor success relied on the subset of T cells selected for CAR generation, with central memory T-cell phenotypes showing greater responsiveness to combination therapy [113].

Ongoing Clinical Trials (refer to the Supplementary Tables S1 and S2): Multiple early-phase clinical trials are actively investigating next-generation CAR-T approaches for neuroblastoma. These include B7-H3 CAR-T trials (NCT04691713, NCT04432649, NCT04897321), GD2 CAR-T with optimized costimulatory domains (NCT02761915), and combination strategies incorporating checkpoint inhibition (NCT05489887) [109]. The integration of safety switches, such as inducible Caspase-9 (iC9), has been incorporated into some constructs to enable rapid elimination of CAR-T cells in case of toxicity [111]. These ongoing studies will provide critical insights into the optimal design, dosing, and combination strategies required to translate the promise of CAR-T therapy into durable clinical benefit for high-risk neuroblastoma patients.

## 6. Discussion

The response of neuroblastoma to immunotherapy is determined by a complex regulatory system composed of four major influencing factors: the TME, tumor cells, the host, and the treatment strategy. The immunosuppressive TME and the low immunogenicity of tumor cells constitute the main intrinsic obstacles, while the host’s genetic background and treatment strategy influence the efficacy of external interventions. The advent of high-resolution technologies like single-cell multi-omics and spatial transcriptomics allows us to precisely dissect the dynamic changes within this system. These discoveries are transforming our view of neuroblastoma from a static disease entity into a dynamic, plastic ecosystem constantly adapting to pressures.

The core challenge now lies in translating this profound understanding into clinical breakthroughs: how to effectively reverse the deeply immunosuppressive TME and ensure that effector immune cells can continuously infiltrate, recognize, and eliminate tumor cells with high heterogeneity and plasticity; and how to predict and intervene in the adaptive evolution of the tumor ecosystem during treatment. Future translational and clinical research may focus on:

### 6.1. Development of Multidimensional Biomarkers and Precision Patient Stratification

The prerequisite for precision medicine is precise prediction. Future efforts need to integrate multi-omics data (e.g., single-cell transcriptomics and epigenomics, spatial transcriptomics, liquid biopsy ctDNA sequencing) to build composite predictive models that comprehensively reflect disease state [114,115]. Such advanced models should systematically incorporate information across multiple dimensions. First, they need to include tumor-intrinsic features, such as mesenchymal state signature scores defined by single-cell data, tumor mutational burden, antigen presentation machinery defect status, and spatial heterogeneity of GD2 expression [56]. Second, they must integrate features of the tumor immune microenvironment, including the abundance of specific immunosuppressive cell subsets (e.g., SPP1+ TAMs, TANs), lineage signatures of T-cell exhaustion or dysfunction, and expression levels of key immune checkpoint molecules (e.g., NECTIN2-TIGIT, B7-H3) [29]. Furthermore, dynamic monitoring of treatment response is crucial, achievable through serial ctDNA monitoring to track clonal evolution, acquired alterations in target antigen-related genes (e.g., GD2 downregulation/loss), and minimal residual disease dynamics during therapy [116]. Finally, models also need to consider host-specific factors, such as Fc receptor polymorphisms and markers related to immune reconstitution capacity [75]. Integrating these multi-layered data via machine learning holds promise for identifying patients likely to have primary resistance before treatment, providing early warning of emerging resistance during therapy, and thereby guiding real-time adjustments to treatment strategies.

### 6.2. Rational Design and Spatiotemporally Sequential Combination Strategies

Given the complexity and adaptability of the neuroblastoma ecosystem, single-agent therapy is insufficient, and combination therapy is imperative. Future combination strategies must be rationally designed based on a deep understanding of resistance mechanisms. These include: combining with drugs targeting TME components, such as using CSF-1R inhibitors or antibodies against the MIF-CD74 axis to deplete/reprogram TAMs [107,117]; combining with agents targeting the physical and metabolic TME (e.g., anti-angiogenics, CSF-1R inhibitors targeting TAMs) to improve immune cell infiltration and function [118,119]; or using IDO inhibitors/adenosine pathway antagonists to reverse metabolic suppression. For overcoming tumor cell-intrinsic resistance, consider combinations with epigenetic modulators (e.g., EZH2 inhibitors, BET inhibitors, HDAC inhibitors) to reverse the MES state, upregulate MHC-I and GD2 expression, and thereby enhance tumor cell immunogenicity and sensitivity to targeted therapy [120]. Combining with metabolic intervention drugs (e.g., CPT1A inhibitors) to attack the fatty acid oxidation pathway that resistant cells depend on [108]. Dynamically adjusting combination regimens based on the evolution of the tumor ecosystem during treatment (e.g., detection of MES-state enrichment, upregulation of specific checkpoints). For instance, sequencing epigenetic drugs or TAM-targeting agents after frontline chemotherapy or CAR-T therapy has cleared the bulk of ADR cells, aiming to eliminate the exposed or induced MES cells and immunosuppressive microenvironment [94].

### 6.3. Development of Next-Generation Engineered Immunotherapy Platforms

Addressing the limitations of current immunotherapies necessitates the development of next-generation platforms. This includes designing dual- or multi-target CARs (e.g., GD2/B7-H3, GD2/ALK) to counteract antigen escape and heterogeneity, providing more treatment options [121,122]. Developing CARs by co-expressing cytokines (e.g., IL-15, IL-7) to enhance persistence, or expressing dominant-negative receptors (e.g., DN TGF-βR) to resist TME suppression [123]. Exploring controllable switch systems (e.g., drug-inducible, logic-gated) in CARs design to enhance safety. Advancing CAR-NK cell therapy into clinical investigation, leveraging its favorable safety profile, potential advantages, and distinct cytotoxic mechanisms [124]. Exploring oncolytic viruses for neuroblastoma, which not only directly lyse tumor cells but also release tumor antigens in situ and stimulate potent anti-tumor immunity [125]. Developing personalized mRNA vaccines based on neoantigens or tumor-associated antigens, aiming to induce or amplify endogenous, polyclonal anti-tumor T-cell responses complementary to existing therapies [126].

## 7. Conclusions

In conclusion, immunotherapy for neuroblastoma has moved from the laboratory to the clinic, growing into an indispensable pillar of standard care for high-risk patients. However, fully unleashing its curative potential requires us to shift from understanding single factors to adopting a systemic perspective—analyzing and intervening in the complex interaction network between tumor, TME, host, and therapy. By integrating cutting-edge multi-omics technologies to reveal dynamic patterns, developing multidimensional biomarkers for precise stratification, designing rational combination and sequential strategies for multi-pathway synergy, and pioneering next-generation immunotherapeutic products to overcome existing bottlenecks, we are moving towards an era of precise, dynamic, and effective immunotherapy for neuroblastoma.

## Data Availability

No new data were created or analyzed in this study.

## Supplementary Materials

The following supporting information can be downloaded at: https://www.mdpi.com/article/10.3390/cells15050441/s1, Table S1: Established Clinical Evidence vs. Emerging Hypotheses in Neuroblastoma Immunotherapy. Table S2: Ongoing Clinical Trials Related to Immunotherapy in Neuroblastoma.

## Abbreviations

The following abbreviations are used in this manuscript:

CAR-T	chimeric antigen receptor T cells
NB	Neuroblastoma
HR-NB	high-risk neuroblastoma
MDSCs	Myeloid-derived suppressor cells
NK	natural killer
ARG-1	arginase-1
ROS	reactive oxygen species
TAMs	Tumor-associated macrophages
TANs	tumor-associated neutrophils
IDO	Indoleamine 2,3-dioxygenase
CAFs	cancer-associated fibroblasts
ADR	adrenergic
MES	mesenchymal
CRC	core transcription regulatory circuitry
SE	super enhancer
MHC-I	major histocompatibility complex class I
ADCC	Antibody-dependent cell-mediated cytotoxicity
αSMA	α-Smooth Muscle Actin
DDR2	Discoidin Domain Receptor Tyrosine Kinase 2
ECM	extracellular matrix
HB-EGF	Heparin-Binding EGF-like Growth Factor
NFAT	Nuclear Factor of Activated T cells
ADCP	Antibody-Dependent Cellular Phagocytosis
